# Craftwell: a feasibility and acceptability study of outdoor heritage crafting for wellbeing and mental health

**DOI:** 10.3389/fpubh.2025.1556230

**Published:** 2025-03-05

**Authors:** Emily Shoesmith, Patricia M. Darcy, Stephanie Piper, Piran C. L. White, Andy Needham, Aimée Little, Gareth Perry, Peter Coventry

**Affiliations:** ^1^Department of Health Sciences, University of York, York, United Kingdom; ^2^Department of Archaeology, University of York, York, United Kingdom; ^3^Department of Environment and Geography, University of York, York, United Kingdom; ^4^York Environmental Sustainability Institute, University of York, York, United Kingdom

**Keywords:** mental health, wellbeing, community, environment, crafting

## Abstract

**Background:**

There has been increasing interest in creativity, heritage and nature to improve health-related outcomes. However, limited research has examined the intersection of heritage crafting in the context of natural spaces. This study aims to explore the feasibility and acceptability of an archaeologically informed outdoor heritage crafting intervention.

**Methods:**

A mixed-methods single group before and after feasibility study was conducted. Participants completed questionnaires, including validated items measuring outcomes related to mental health, wellbeing, social connectedness, mindfulness, perceived state of flow and the connection with nature and the environment. Qualitative interviews were conducted with participants to explore their experiences, and data were analyzed using thematic analysis.

**Results:**

Forty-eight participants from a University in the United Kingdom attended the workshops, achieving the recruitment target within the required timeframe. The response rate to all pre-workshop measures was 100% and remained at 100% post-workshop, with the exception of missing data for two participants (4.2%) for measures assessing wellbeing and anxiety, and missing data for five participants (10.4%) for the measure assessing depression. Therefore, response and retention rates indicate high levels of feasibility to conduct a robust evaluation of this intervention. Five themes were identified, including: participant motivation to sign up; engaging with creative activities in a natural setting; skilled facilitation and a flexible approach; group delivery, and duration and frequency of workshops. Overall, the workshops were positively received by participants, primarily attributing their satisfaction to engagement with group-based creative activities in a natural setting with an educational component.

**Conclusion:**

This study has shown it is feasible and acceptable to deliver and evaluate an archaeologically informed heritage crafting intervention to support wellbeing. These results suggest the need for formal testing of the potential health benefits of the intervention to address policy imperatives for developing and implementing community- and place-based approaches to support mental health.

## 1 Introduction

Mental health disorders are the third leading cause of years lived with disability, with a global prevalence of greater than 10% (1). However, mental health is not solely the absence of illbeing, it is also the presence of wellbeing (2), both hedonic (e.g., happiness) and eudaimonic (e.g., having meaning in life) (3, 4). Globally, wellbeing is an important topic for individuals, societies and public policy (5). It is increasingly being viewed as an important component for policy makers due to its impact on economic, health, social and cultural aspects of life (6). The variety of perceptions of what is meant by ‘positive wellbeing’ indicates that it is imperative to explore alternative ways of addressing quality of life and wellbeing for the population, at all stages of life and levels of health (5). Focus is increasingly turning toward the important role that both creativity (7) and the natural environment (8, 9) play in maintaining and enhancing mental health and wellbeing. Creative projects can serve as valuable community-based assets to support people to meet their health and wellbeing needs, but are difficult to sustain despite their impact (10). There is a need for substantial and sustained investment in new approaches that can enhance capacity and access to community, place-based mental health solutions.

It is well-established that creative activities contribute to positive development and wellbeing of a range of populations (11–14). The opportunity to participate in preferred creative activities may increase wellbeing and life satisfaction, while potentially decreasing psychological discomfort, depression, and anxiety (15). For example, existing evidence has reported engaging in craft activities can promote a sense of sharing and belonging (16, 17), offer a distraction from emotional stress by creating feelings of relaxation (17, 18), and provide a healing and protective effect on mental wellbeing (19). In evaluating the impact of crafting on wellbeing, literature frequently refers to the concept of flow (20), and mindfulness (21, 22). Flow refers to an intense involvement in an activity within which the person experiences meaningfulness, which may subsequently lead to positive outcomes (15, 23). Mindfulness is defined as a state of ‘nonjudgemental moment-to-moment awareness’ (24), and has the potential to regulate stress and improve cognitive and emotional functioning (25). Mindfulness may work synergistically with creativity (26). Research has indicated that experiential and social aspects of crafting fosters components of both creativity and mindfulness, through which craft can contribute to emotional satisfaction, social connection, personal agency, and overall wellbeing (22). Additionally, there is evidence that people who engage in heritage crafting activities expressed a connection to cultural tradition (27, 28). This perceived link between crafting and tradition provides the opportunity for heritage, crafting and wellbeing to work in tandem.

Intangible cultural heritage refers to the various practices, performances and expressions, knowledge systems, skills, crafts, and traditional culture that are inherited by various groups (29). Traditional handmade intangible cultural heritage projects involve skills handed down from previous generations of continuous development, and include a wide range of content, such as weaving, embroidery and ceramic making (30). There has been increasing evidence for links between craft heritage activities and wellbeing (31), and having access to these activities has been shown to enhance wellbeing (32–35). A growing body of literature indicates that craft heritage, including written heritage (36), material culture (37, 38), rock art (39), and ancient architecture (40), is advantageous for contemporary wellbeing (41). These forms of heritage crating offer diverse groups of individuals opportunities to engage in enriching activities that foster learning, tactile interaction, creativity, and storytelling.

In addition to creative activities, connection with and activities in natural environments can also play a critical role in maintaining and enhancing mental health and wellbeing (8, 9, 42, 43). Existing theories, such as the attention restoration theory (ART) (44) and the stress restoration theory (SRT) (45) suggest that contact with nature can influence both productivity and wellbeing. To recover from stressors and demands of daily lives, individuals need to replenish lost resources by engaging in activities that restore old resources or generate new resources (46). Both ART and SRT propose that exposure to nature is able to restore these emotional and cognitive resources, subsequently reducing levels of stress. Engagement with nature provides the opportunity to capture involuntary attention and provide relief from everyday stressors and demands (47). People who have higher levels of nature connectedness tend to have higher levels of both hedonic (48) and eudaimonic (9) wellbeing. Additionally, evidence suggests that community- and place-based participatory art practices may enhance subjective wellbeing, promote social cohesion, and strengthen community networks (49). These benefits may be further amplified when activities are culturally rooted (50, 51).

Given the salutogenic characteristics of creative tasks and engagement with outdoor natural spaces, we developed the means to pilot test the likely mental health and wellbeing benefits of an outdoor heritage crafting intervention known as Craftwell. Craftwell involved two workshops: (1) crafting replica Anglo-Saxon pots, or (2) crafting Mesolithic beads. This mixed-methods study aimed to assess the feasibility and acceptability of outdoor craft heritage workshops. Specifically, the research questions were:

1. Are outdoor craft heritage workshops feasible to deliver and evaluate (including recruitment rate, attrition rate, data collection procedures)?
2. What are participant perceptions of the outdoor craft heritage workshops?

## 2 Methods

### 2.1 Study design

A mixed-methods single group before and after feasibility study to explore outdoor heritage craft workshops delivered to a student population.

### 2.2 Setting and participants

This study was conducted with adult students (18 years+) at the University of York, United Kingdom (UK), who were registered on undergraduate or postgraduate programmes at the time of the workshops (spring and summer term 2022–2023). The recruitment of student volunteers is a common example of convenience sampling for feasibility studies (52).

Students in the Department of Archaeology who had already completed a course of study that involved taking part in crafting activity at the York Experimental Archaeological Research (YEAR) Centre were not eligible.

### 2.3 Overview of workshops

**Figure 1:**
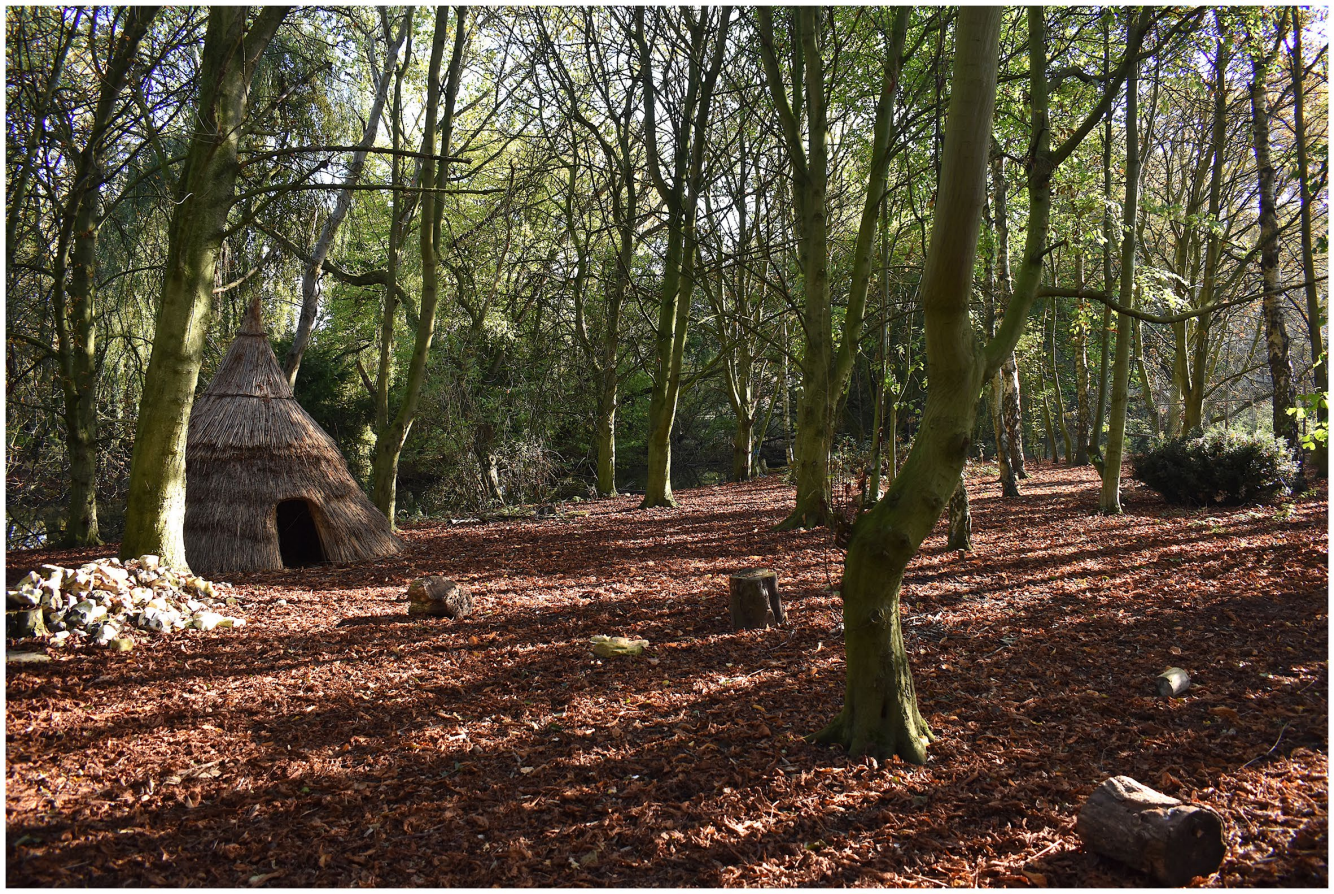
Image of the YEAR Centre.

The YEAR Centre is an outdoor space on campus used for teaching and researching experimental archaeology. It is located in a quiet area of woodland by a lake. In this space, there are reconstructions of a prehistoric house and a Viking workshop, as well as open structures with central hearths that provided shelter and warmth during the workshops (see Figure 1). The workshops were delivered by an archaeologist with extensive experience and expertise in engaging archaeology with the wider community.

The workshops took place between 8th March 2023 and 7th June 2023, offered once weekly on a Wednesday afternoon (14:00–16:00) during spring and summer term times (10 workshop dates available in this duration). A minimum of 4 and a maximum of 12 participants were invited to each workshop.

#### 2.3.1 Bead making workshop

In the bead making workshop, participants recreated stone beads using the materials and techniques that archaeologists think people used 11,000 years ago. The beads emulated findings from the Mesolithic site of Starr Carr, North Yorkshire, where evidence for a bead making workshop has been found (53–55). The beads were made from a soft shale collected from the Yorkshire coast, which is the most likely source used by people at Star Carr (55). The holes were hand-drilled using specially made flint drill tips, known as awls, which have also been found at Starr Carr, with microscopic traces of wear suggesting they were used in this way (53). The workshops gave participants the opportunity to engage with prehistoric craft making using locally sourced materials, and explore different methods of drilling, decorating, and stringing the beads. To string them, participants recreated simple twisted string made from plant fibre, which has also been recovered from archaeological sites dating to the same period.

#### 2.3.2 Pottery workshop

In the pottery workshop, participants made pots based on Anglo-Saxon types used during the 5-6th Century CE (56, 57). Pots of distinctly early Anglo-Saxon type are found on sites throughout eastern England. These vessels were used for a range of activities including cooking, storage, brewing, dairying and as containers to hold the cremated remains of the dead (58). Anglo-Saxon potters made their wares by hand, using coils of clay to build up the vessel wall, and these were fired in simple bonfires. Although most pots were plain, those used for brewing, drinking and as cremation urns were highly decorated with stamp impressions and incised lines (59). Participants used the same techniques to achieve their pots. Examples of decorative patterns that have been discovered on Anglo-Saxon pots were available for participants to recreate on their own pots.

### 2.4 Measures

A questionnaire was developed by a multi-disciplinary team of academics. The questionnaire included validated items, as detailed below.

#### 2.4.1 Demographic data

Demographic data were collected about participants’ age (in bands), gender (male/female/non-binary), ethnicity, presence of a mental or physical health condition, and the extent to which these limit day-to-day activities (limited substantially/not limited substantially/not limited), stage of degree (undergraduate year 1, 2, or 3, master’s degree, or PhD), and name of degree.

#### 2.4.2 Mental health and wellbeing

The Short Warwick Edinburgh Mental Wellbeing Scale (SWEMWBS) (60), the PHQ-8 (61), and the GAD-7 (62) were included. The PHQ-8 is an eight-item version of the PHQ-9 (63) that omits the item assessing the risk of suicide. As such, this measure is commonly used in non-clinical populations. Higher scores on the SWEMWS represent better wellbeing, whereas higher scores on the PHQ-8 and GAD-7 represent higher depression and anxiety, respectively. These measures were used to collect data pre- and post-workshop.

#### 2.4.3 Social connectedness

##### 2.4.3.1 Loneliness

The 3-item short version of the UCLA loneliness scale (64) was included to collect data pre- and post-workshop, and higher scores on this scale represent greater loneliness.

##### 2.4.3.2 Social connectedness

The Social Connectedness Scale-Revised (65) was included to assess the degree to which participants felt connected to others in their social environment pre- and post-workshop. Participants were asked to indicate their agreement with 20 statements on a 6-point Likert scale (1 = strongly disagree; 6 = strongly agree). Negatively worded items (e.g., ‘I feel like an outsider) were reverse coded, and items were calculated to generate a total score (1–120), with higher scores representing more connectedness to others.

##### 2.4.3.3 Social inclusion

The Social Inclusion Scale (66) incorporates three subscales measuring social isolation, relations, and acceptance. Participants were asked to indicate their agreement with 21 statements on a 4-point Likert scale (1 = not at all; 4 = definitely). Negatively worded items (e.g., I have felt terribly alone and isolated’) were reverse coded, and items were calculated to generate a total score overall (1–84), with higher scores representing a higher perception of social inclusion. This measure was used to collect data pre- and post-workshop.

#### 2.4.4 Nature and environment

##### 2.4.4.1 Nature connectedness

The Nature Connection Index (NCI) (67) was included. Participants were asked to indicate agreement to six statements on a 6-point Likert scale (1 = completely disagree; 6 = strongly agree). Items included statements such as: ‘I always find beauty in nature’ or ‘I always treat nature with respect’. To score this scale, a conversion spreadsheet was available to calculate a total Nature Connection Index score from the individual items, with higher scores representing higher connection to nature. This measure was used to collect data pre- and post-workshop.

##### 2.4.4.2 Time spent outdoors

A question from the Natural England People and Nature Survey (GOV.UK., 2020) was included to ask participants about occasions in the last 12 months when they spent time outside (e.g., open spaces in and around towns and cities including nature areas). Participants were asked to indicate how often, on average, they had spent time outdoors, ranging from every day to less often than 2–3 months. This question was asked pre- and post-workshop.

##### 2.4.4.3 Engaging with environment

Participants were asked whether they engaged regularly in recycling, buying eco-friendly products, buying seasonal or locally grown food, opting to walk or cycle rather than using a car, encouraging people to protect the environment, engaging in environmental or conservation organizations, volunteering to help care for the environment, or donating money to support an environmental or conservation organization.

#### 2.4.5 Mindfulness

The Mindfulness Attention Awareness Scale (MAAS) (68) was included. Participants were asked to indicate agreement to 15 statements on a 6-point Likert scale (1 = almost always; 6 = almost never). Items included statements such as: ‘I do jobs or tasks automatically, without being aware of what I’m doing’, ‘I find myself preoccupied with the future or the past’, and ‘I find myself doing things without paying attention’. To score this scale, an average of the 15 items is calculated. This measure was used to collect data pre- and post-workshop.

#### 2.4.6 Perceived state of flow

The Flow Short Scale (69) was included in the post-workshop questionnaire only to measure the components of flow experience. Participants were asked to indicate agreement to 13 statements on a 3-point Likert scale (1 = not at all; 3 = very much). Items included statements such as: ‘I feel just the right amount of challenge’, and ‘I am totally absorbed in what I am doing’. The last item ‘I am worried about failing’ was reverse coded, and items were calculated to generate a total score (1–39), with a higher score representing a higher perceived sense of flow.

#### 2.4.7 Experience of the workshop setting

The Perceived Restorativeness Scale (PRS) (70) was included in the post-workshop questionnaire only to evaluate the participant’s experience in the setting. Participants were asked to indicate agreement to 26 items on a 7-point Likert Scale (0 = not at all; 6 = completely). Items included statements such as: ‘being here is an escape experience’, and ‘the setting is fascinating’. Negatively worded statements (e.g., ‘this place is boring’) were reverse coded, and items were calculated to generate a total score (0–156), with a higher score representing better experiences.

Lastly, interviews were conducted with a sub-sample of participants. A semi-structured topic guide was developed by the research team to explore their experiences of taking part in the workshops (including the signing up process and completion of questionnaires). The full topic guide is presented in Supplementary Material 1.

### 2.5 Recruitment and procedures

Flyers were distributed to promote the workshops via social media (e.g., X), departmental bulletins, and announcements via the University of York Student’s Union webpages. The flyers included a QR code to take potential participants to an Expression of Interest form using a Google form. This form invited potential participants to confirm they were a student at the University of York and to select their preferred crafting activity (pottery making or bead making) and preferred dates for taking part.

Eligible participants were then contacted by a member of the research team to invite them to a workshop. This invitation included a personalized link, generated through Qualtrics (Qualtrics, Provo, UT) questionnaire software, for participants to complete a pre-workshop questionnaire before taking part in the study. All participants could complete the pre-workshop questionnaire within a two-week period before the workshop started. Participants were also directed to an embedded participant information sheet, a risk assessment summary, and a consent form. Consent to participate was indicated by ticking an online check box. Participants could also tick an optional consent box to participate in follow-up interviews. At the beginning of the questionnaire, each participant was assigned an ID number to anonymise all data. All pre-workshop data were stored on the Qualtrics server at the University of York until all workshops were completed.

Immediately after completion of the workshop, participants were required to complete a post-workshop questionnaire in-person. The data were inputted into Qualtrics by a member of the research team, using the same ID numbers generated during the pre-workshop questionnaire to match participant responses across timepoints. The post-workshop data were stored on the Qualtrics server at the University of York until all workshops were completed.

Following completion of all workshops, a purposive sampling approach was used to select a sub-sample of participants for interviews based on their age, gender, stage of degree, and the crafting activity attended. Participants were invited to interview via email, and interviews were conducted via Zoom. Interviews were audio-recorded and transcribed verbatim. Transcripts were assigned with the participant ID numbers to anonymise the transcripts.

Ethics approval was granted by the Department of Archaeology at the University of York on 20^th^ February 2023.

### 2.6 Data analysis

Descriptive summary statistics are presented for demographic variables. The raw dataset was used to calculate mean scores, ranges, and standard deviations (SD) for outcome variables at the two timepoints, as is convention with feasibility studies (71). These descriptive statistics were calculated using SPSS version 28.0 (IBM®).

For qualitative data, thematic analysis was conducted and adopted a deductive *a priori* template of codes approach, which allows for codes to be applied as a means of organising text for subsequent information. Data were interrogated to extract meaningful data segments following the code template. Transcripts were systematically coded, and codes with similar content were collated into preliminary themes. Two authors (ES and PD) independently reviewed the themes to ensure consensus with the assignment of themes and illustrative quotes.

## 3 Results

**Figure 2:**
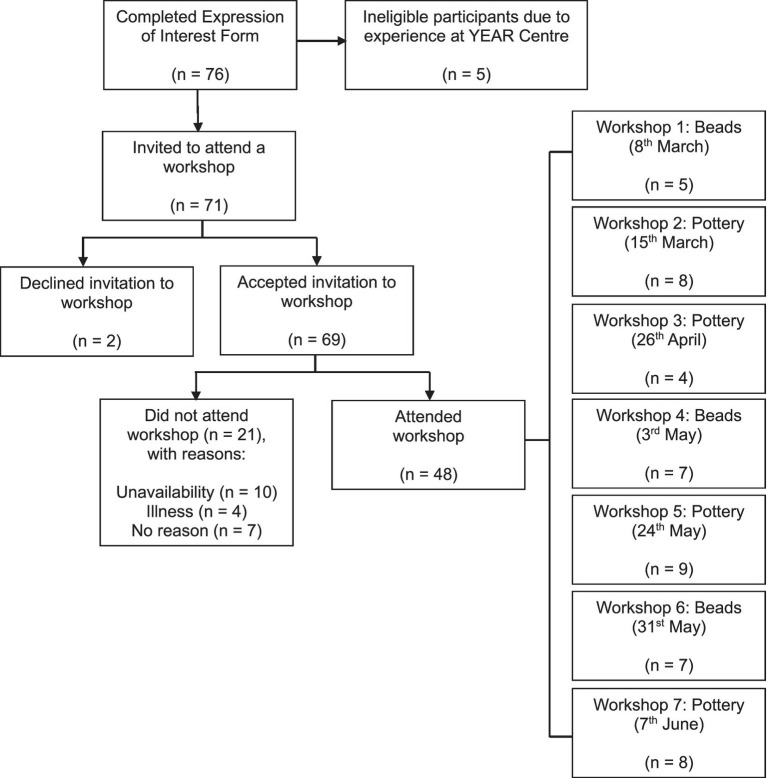
Flow diagram of recruitment, workshop attendance and non-attendance.

Seventy-six participants completed the Expression of Interest form. Of these, five were ineligible as they had previous experience at the YEAR Centre. Therefore, 71 eligible participants were invited to attend a workshop, of which 69 (97.2%) initially accepted. Forty-eight participants (69.6%) attended across four pottery and three bead making workshops; 21 (30.4%) consented participants did not attend. Reasons for non-attendance included illness (*n* = 4) and unavailability due to lectures or exams (*n* = 10); seven participants did not provide a reason (Figure 2).

Group size varied, with an average of seven participants per workshop (range = 4 to 9; Figure 2). Twenty-nine participants (60.4%) attended pottery workshops, and 19 participants (39.6%) attended bead making workshops. A summary of participant characteristics is presented in Table 1.

**Table 1:** Participant characteristics (*n* = 48).

Characteristics		% (*N*)
Gender	Female	68.8 (33)
	Male	22.9 (11)
	Non-binary	8.3 (4)
Age	18–24	54.2 (26)
	25–34	33.3 (16)
	35–44	8.3 (4)
	45–54	2.1 (1)
	55–64	2.1 (1)
Ethnicity	British	64.6 (31)
	Other white ethnic group	12.5 (6)
	Chinese	10.4 (5)
	Other	4.2 (2)
	Indian	2.1 (1)
	Pakistani	2.1 (1)
	Irish	2.1 (1)
	Any other Asian background	2.1 (1)
Stage of degree	Undergraduate Year 1	8.3 (4)
	Undergraduate Year 2	10.4 (5)
	Undergraduate Year 3	20.8 (10)
	Postgraduate masters	37.5 (18)
	PhD	22.9 (11)
Degree department	Medieval studies	33.3 (16)
	Environment and geography	25.0 (12)
	English and related literature	22.9 (11)
	History/History of art	12.5 (6)
	Medical/Health sciences	6.3 (3)
Day-to-day activities limited due to a mental or physical health condition	Yes – limited substantially	4.2 (2)
	Yes – but not limited substantially	20.8 (10)
	No	75 (36)
Mental or physical health condition	Mental health condition such as depression or anxiety	16.7 (8)
	Any other long-term illness or condition	10.4 (5)
	Dyslexia or an autistic spectrum disorder	6.3 (3)
	A learning difficulty/disability or cognitive impairment (e.g., Down’s syndrome)	2.1 (1)
	None reported	64.6 (31)

### 3.1 Evaluating feasibility: quantitative data

#### 3.1.1 Mental health and wellbeing

There were no missing data for measures assessing depression, anxiety, and wellbeing pre-workshop. For the post-workshop questionnaire, there were missing data for two participants on the GAD-7 and SWEMWBS measures, and missing data for five participants on the PHQ-8 measure (Table 2).

**Table 2:** Mean scores, ranges, and standard deviations for depression, anxiety, and wellbeing scores pre- and post-workshop.

	Depression score		
	Mean	Range	Standard deviation
Pre-workshop (*n* = 48)	16.1	8–32	5.6
Post-workshop (*n* = 43)	16.7	9–28	5.0

	Anxiety score		
	Mean	Range	Standard deviation
Pre-workshop (*n* = 48)	14.1	7–28	5.4
Post-workshop (*n* = 46)	14.3	8–27	4.8

	Wellbeing score		
	Mean	Range	Standard deviation
Pre-workshop (*n* = 48)	22.4	13–32	4.1
Post-workshop (*n* = 46)	22.9	13–34	4.5

#### 3.1.2 Social connectedness

There were no missing data for loneliness, social connectedness, or social inclusion measures at either timepoint (Table 3).

**Table 3:** Mean scores, ranges, and standard deviations for loneliness, social connectedness, and social inclusion scores pre- and post-workshop.

	Loneliness score		
	Mean	Range	Standard deviation
Pre-workshop (*n* = 48)	6.2	3–9	1.9
Post-workshop (*n* = 48)	5.7	3–9	1.8

	Social connectedness score		
	Mean	Range	Standard deviation
Pre-workshop (*n* = 48)	77.3	36–110	19.4
Post-workshop (*n* = 48)	84.1	42–113	17.4

	Social inclusion overall score		
	Mean	Range	Standard deviation
Pre-workshop (*n* = 48)	61.9	42–81	9.4
Post-workshop (*n* = 48)	62.3	42–80	9.2

#### 3.1.3 Mindfulness

There was no missing data for the MAAS at either timepoint. The mean score for mindfulness pre-workshop was 3.6 (range = 2–5; SD = 0.82), and the mean score for mindfulness post-workshop was 3.4 (range = 1–5; SD = 0.86) (−0.2).

#### 3.1.4 Nature and environment

There was no missing data for nature connectedness at either timepoint. The mean Nature Connection Index score pre-workshop was 24.9 (range = 5–34; SD = 7.9), and the mean Nature Connection Index score post-workshop was 25.3 (range = 1–34; SD = 8.5).

For time spent outdoors, six (12.5%) participants indicated they spent time outside every day both pre- and post-workshop. Thirty-three participants (68.8%) reported during the pre-workshop questionnaire that they spent time outdoors once a week or more, but not every day, compared with 35 (72.9%) participants post-workshop. Only nine (18.7%) participants reported that they spent time outdoors less than this (e.g., once or twice a month, once every 2–3 months or less) pre-workshop, compared to seven (14.6%) participants post-workshop.

The majority of participants engaged in at least one environmental activity pre-workshop (Table 4), with the most common being recycling (93.8%, *n* = 45), and the least frequent being donating money to an environmental or conservation organization (6.25%, *n* = 3).

**Table 4:** Engagement with environmental activity.

Environmental activity	Pre-workshop % (n)
Recycling items	93.8% (45)
Buying eco-friendly products and brands	52.1% (25)
Buying seasonal or locally grown food	31.3% (15)
Walking or cycling instead of using car	83.3% (40)
Encouraging people to protect the environment	70.8% (34)
Member of environmental or conservation organisation	18.8% (9)
Volunteering to help care for the environment	16.7% (8)
Donating money to environmental or conservation organisation	6.25% (3)

#### 3.1.5 Perceived sense of flow and experience of the workshop setting

There was no missing data for the Flow Short Scale or PRS. The mean score for perceived sense of flow was 25.9 (range = 19–34; SD = 3.8), and the mean score for the PRS was 124.6 (range = 71–156; SD = 18.5). These scores indicate participants experienced a high sense of flow and positive experience of the workshop setting.

### 3.2 Exploring feasibility and acceptability of the workshops: qualitative data

Interviews were conducted with 15 participants. Of these, 10 attended the pottery making workshop and five attended the bead making workshop. Participant characteristics for those completing interviews are presented in Table 5. The interview durations ranged from 24 min and 32 s to 89 min and 20 s (average = 44 min and 36 s).

**Table 5:** Participant characteristics for interview sub-sample (*n* = 15).

Characteristics		**% (N)**
Gender	Female	73.3 (11)
	Male	20 (3)
	Non-binary	6.7 (1)
Age	18–24	40 (6)
	25–34	33.3 (5)
	35–44	26.7 (4)
Ethnicity	British	60 (9)
	Other white ethnic group	26.7 (4)
	Chinese	6.7 (1)
	Other	6.7 (1)
Stage of degree	Undergraduate Year 1	6.7 (1)
	Undergraduate Year 2	6.7 (1)
	Undergraduate Year 3	20 (3)
	Postgraduate masters	33.3 (5)
	PhD	33.3 (5)
Degree department	Medieval studies	20 (3)
	Environment and geography	20 (3)
	English and Related literature	20 (3)
	History/History of art	20 (3)
	Medical/Health sciences	20 (3)
Day-to-day activities limited due to a mental or physical health condition	Yes – limited substantially	6.7 (1)
	Yes – but not limited substantially	26.7 (4)
	No	66.7 (10)
Mental or physical health condition	Mental health condition such as depression or anxiety	26.7 (4)
	Any other long-term illness or condition	6.7 (1)
	Dyslexia or an autistic spectrum disorder	13.3 (2)
	A learning difficulty/disability or cognitive impairment (e.g., Down’s syndrome)	6.7 (1)
	None reported	46.7 (7)

Thematic analysis of interview data resulted in the identification of five themes, including: (1) participant motivation to sign up; (2) engaging with creative activities in a natural setting; (3) skilled facilitation and a flexible approach; (4) group delivery, and (5) duration and frequency of workshops. To illustrate themes, quotations are presented verbatim, with the gender, age range and degree level of participants provided in brackets.

#### 3.2.1 Participant motivation to sign up

When asked about their primary reason for signing up for the workshops, most participants cited the appeal of engaging in creative activities. They found these activities therapeutic and a good distraction from daily stress. Additionally, one participant mentioned that the high cost of such activities often creates a barrier, despite their potential benefits.

*“Clay making, I was like, that’s for me, especially going to university with all the stress and being busy, I didn’t find time to do art, and I do find it quite therapeutic. So, I thought, that’s a great time, I will actually do it instead of just thinking about it.”* (Female, 18-24yrs, Undergraduate Year 3)

*“I really like pottery and ceramics, and it’s actually very expensive to do it outside of a study, and yeah, I just thought the chance to be able to do that would be great.”* (Female, 25-34yrs, PhD)

In addition to engaging with creative activities, some participants acknowledged that the workshop offered a unique, creative task that was historically framed, providing the opportunity to try something new in addition to learning more about heritage crafting.

*“It was something which I’ve not really done before, so yeah, it just seemed an interesting activity to go and give it a try, especially because it was framed in a historical context of Anglo-Saxon pottery, it was a bit more unique.”* (Male, 18-24yrs, PhD)

*“I very much appreciated the opportunity to have hands-on experience of archaeology and of practices, really, into archaeology. So, the idea of this being able to, sort of, learn how to make stone beads, well, that was all I really needed to become interested. I just like the idea of being able to do things hands-on and experience archaeology in new ways.”* (Female, 18-24yrs, Postgraduate Masters)

One of the key reasons participants signed up for the workshop was the chance to engage in creative activities outdoors rather than indoors.

*“I think actually, no, I wouldn’t have had the same motivation to sign up if it was an indoor activity, I think it was definitely a pretty big incentive that it was outdoors and that was another thing my flat mate said, it’s so peaceful, you sit around a little fire, it’s great.”* (Female, 18-24yrs, Undergraduate Year 2)

Lastly, some participants expressed that no single factor motivated them to sign up for the workshop. Instead, a combination of interacting factors appealed to them, which they believed would offer them a more holistic experience.

*“It works on more than one level, that sort of thing, being out in the environment in the fresh air with other people and then focusing on a thing and creating something. Yes, there’s all these different things at play and I just thought, yes, I’m fascinated, I want to know more, and I want to have a go myself.”* (Female, 35-44yrs, PhD)

#### 3.2.2 Engaging with creative activities in a natural setting

The majority of participants described the setting for the workshops as enjoyable and relaxing, and highlighted the value of being in an outdoor space that felt like a novel part of an otherwise familiar campus.

*“It was pretty enjoyable. I went to the part of the university that I haven't been before actually, so it was nice being in nature and I loved the environment around all the trees and quiet there. So, that was nice sitting by the fire and, yeah, it's pretty enjoyable and relaxing.”* (Female, 18-24yrs, Undergraduate Year 3)

*“I really liked the setting; it was really nice. It was very relaxing, and you kind of felt like you were sort of tucked away and isolated from the rest of uni, even though we were literally right next to it, but that was nice. And then the fire was very nice. And yeah, I mean, it was nice just to slow down and take a pause and do something a bit creative for a few hours.”* (Female, 25-34yrs, PhD)

Participants frequently mentioned elements of the natural environment that appeared to enhance their experiences of the workshop and promote wellbeing. These included the greenery of the location and indirect interaction with wildlife around them.

*“I think it was the fact you could hear the birds, there was the odd squirrel running around, you could see down to the lake. Yes, it’s just nice to feel like… I think it’s particularly having the birdsong around just makes it that bit more relaxing”* (Female, 25-34yrs, PhD)

*“It feels very enclosed by trees, I think, yeah, the tree surroundings, especially as they’re right next to the stray* [area of historic common land] *as well, it takes you right out of the city or the town, and it felt quite sheltered and relaxed.”* (Male, 35-44yrs, Undergraduate Year 3)

Despite these benefits, participants noted the limited surface space available outdoors made it challenging to engage with creative activities.

*“The only thing would be the lack of, like, workspace. You know, like I say, people sometimes just had to hold the thing they were making, or I knelt down and did it, I found a piece of wood and I did it on a piece of wood, kind of thing. That was a little bit challenging, and it probably limited by ability to do as good as I could have done. Maybe that's one thing I would have changed, yeah, just something better to use to actually work on.”* (Female, 55-64yrs, Postgraduate Masters)

For participants who had a physical disability or health condition, sitting on a log or having an unstable work surface could be uncomfortable or challenging. Specific techniques which required dexterity and fine motor skills were also experienced as challenging.

*“Yes, I didn’t use too many of them [tools], I think, but because I think I knew that it would be a little bit fiddly for me, it was sort of the issue of not necessarily having a flat surface to work on and also trying to hold the thing and do something else with another hand, and I thought I’m not going to be very good at that, and I’ll probably end up dropping it.”* (Female, 35-44yrs)

*“So, it was a difficult process of doing it. It was a bit fiddlier, and the constant twisting, this is just a me issue, I’ve got bad joints, so the twisting of the flint, that was just something, that was to do with me. But it wasn’t unenjoyable because of it, and I just swapped hands.”* (Non-binary, 18-24yrs)

However, one participant highlighted that altering the environment to facilitate the creative activity would have detracted from the importance of connecting with the past.

*“The only thing is not having a stable space to work on and being sat on a log, but again I understand why it was like that, that was the point of it. And if you had then suddenly brought in pillows and cushions, and clipboards, it probably would have detracted from that immersion in the past and what you’re supposed to be achieving. So, it wasn’t something that I didn’t like, you know? It just was slightly awkward and uncomfortable at times, but we all made do, you know? It was fine.”* (Female, 35-44yrs, PhD)

Lastly, some participants mentioned that their experience of engaging with crafting activities outdoors was particularly beneficial due to the time of year, and this may have been different if the weather was inclement. Whilst most participants felt that the fire enhanced the atmosphere, some mentioned that the smoke from the fire interfered with their crafting.

*“The weather was beautiful, there were no mosquitoes or anything, so, yeah, it was great. But I know it would probably be significantly different if it was raining, or it was cold because of course then it's a bit uncomfortable.”* (Female, 18-24yrs, Undergraduate Year 3)

*“The only thing is the wind, but that’s impossible to manage, because all the fire, you know, the smoke, it started to come to our eyes, but that’s the only thing that I could find a challenge.”* (Female, 35-44yrs, Postgraduate Masters)

#### 3.2.3 Skilled facilitation and a flexible approach

Participants appeared to suggest that skilled and knowledgeable facilitation was a key contextual feature that enhanced their workshop experience, citing that the educational element was as valuable as the creative hands-on activity.

*“Going round the actual site and the things that were there, which was fab, like having a nosey and the [facilitator] was very open and let us look around and ask loads of questions and stuff and were very knowledgeable. It was just a really interesting experience.”* (Female, 35-44yrs, PhD)

Additionally, many participants appreciated the facilitator’s flexible approach. In both the bead making and pottery workshops, this flexible approach fostered a sense of autonomy, allowing participants to craft unique products aligned with their personal preferences and apply newly acquired skills right from the start of the session.

*“I liked that I didn’t feel any pressure to create anything in particular. I knew that we’d been given parameters in the sense that we’d been given information about what clay and pots and tools had been used for in the past, but we didn’t have to recreate that, and I don’t think anyone had the sense of any pressure or anything, which I liked. And there was definitely a sense of, like, being inspired by past and present in terms of what we’d been told about the past but also the environment, so that was nice.”* (Female, 18-24yrs, Undergraduate Year 2)

Furthermore, some participants expressed curiosity about their peers’ creations, adding to the overall intrigue of the experience.

*“I enjoyed the bit where we were given a sharper or a thinner bit of the flint when we got to carve the lines and the actual physical personality and design into the little shale pieces. That was a fun bit because we all did different things, and I liked to see what everybody did. Especially because they were examples.”* (Non-binary, 18-24yrs, Undergraduate Year 1)

#### 3.2.4 Group delivery

Participants commonly expressed a preference for engaging in a group rather than a one-to-one workshop, suggesting that the group format was a key enabler to their participation. They frequently cited the sense of peer support from the new social group and the chance to share experiences with others as reasons for this preference.

*“It was all just really nice to talk to people who were from different departments and realise that other people were interested in completing that activity, so yes, it just got me out of my usual social circle.”* (Female, 18-24yrs, Undergraduate Year 2)

*“I think [group activities] are fairly important. I think that common activity is one of the best ways to bond with people generally, so it’s fairly important to have that available.”* (Female, 18-24yrs, Postgraduate Masters)

The majority of participants attended the workshop independently and mentioned this was beneficial as they could meet new people and concentrate on their own activity.

*“I think it did impact my experience of the workshop because when you take opportunities like that by yourself, in my experience, it’s easier to, sort of, approach that community feeling.”* (Female, 18-24yrs, Postgraduate Masters)

Additionally, some participants mentioned that attending with a peer they already knew may act as a distraction to the task at hand.

*“If I had gone with a friend, it would be mostly an experience that I would be experiencing between me and my friend, and less between me and the group.”* (Female, 18-24yrs, Postgraduate Masters)

*“I was quite happy to go alone and meet new people really. I think, because of the way that the workshop was framed to me, it was more something to go along and, as sort of a solo activity and meet other people there. And I felt if I had taken friends along, it might have distracted a bit more from the overall experience.”* (Male, 18-24yrs, PhD)

#### 3.2.5 Duration and frequency of workshops

The majority of participants indicated that the duration of the workshop was appropriate. There was consensus that a shorter duration would not have allowed sufficient time to complete the activity, yet a longer duration may have restricted uptake due to individual time constraints.

*“Yes, I think it was. If anything, we were all going, can we do another one, and then going, actually, no, we really ought to get back to our desks. And I think if it had been less…if it had been more than two hours, you'd have probably ended up with less signups, we’d be going, I can’t really afford that time. But actually, once you're there, you're going, this is really fun, we want to just keep grinding stones. So, yes, I think two hours was a good amount of time, actually, yes.”* (Female, 25-34yrs, PhD)

Additionally, participants noted that the two-hour workshop struck an ideal balance. It provided educational material at the beginning to offer archaeological context for the crafting, while also allowing ample time for the practical creative activity.

*“I’d say I enjoyed the combination. I think it was nice to have that introduction first and overview, before we got going, rather than just launching straight into the pottery making without any context.”* (Male, 18-24yrs, PhD)

As well as there being a consensus that the duration was appropriate, there was further evidence that offering a series of workshops rather than a one-off event would be desirable. Some participants discussed the potential of the workshop in the context of social prescribing.

*“Multiple exposure, I think, is better, I think I would have preferred to do it more times and be exposed more to the activities and the environment and things, over a period, rather than once.”* (Female, 25-34yrs, PhD)

*“I think it could end up being a really good social prescribing intervention that people could be referred to, even if it is that one-off session, I think it will be beneficial, but it would also be great as a session, like a series of sessions.”* (Female, 35-44yrs, PhD)

While there was a consensus that offering a series of workshops could be beneficial, participants who lived off-campus suggested that the demands of travel to the centre could be a barrier to regular attendance.

*“I just don’t know how personally whether I would have been able to because I think it would have been quite tiring for me. However, was it something nearby? Yes, I definitely think I would attend six different sessions on various different crafty Anglo-Saxony Stone Age things, I would definitely give it a go because I think it would be a lot of fun. But yes, travelling to York six weeks in a row? Probably not.”* (Female, 35-44yrs, PhD)

To address the challenge of commuting for off-campus residents, one participant suggested that the facilitator could direct students to comparable activities nearby.

*“I think something which would be useful actually, was if the workshop was able to then, at the end of the workshop there was a signpost way to find out more about those kinds of things.”* (Male, 18-24yrs, PhD)

## 4 Discussion

This study explored the feasibility and acceptability of offering a novel intervention that combined heritage crafting in an outdoor setting with natural features for mental health and wellbeing. The findings offer preliminary evidence that these workshops were feasible to deliver, as the study achieved its recruitment target within the required timeframe and had high uptake across two terms despite variable weather. We also demonstrated high levels of feasibility to undertake a robust evaluation of the health benefits of this intervention, as we collected a range of demographic and outcome-based data, with high retention of data across two timepoints. These findings highlight these tools and metrics have high face validity and acceptability. Additionally, the workshops were positively received with participants citing satisfaction with the opportunity to engage in an outdoor creative task that had educational value, provided for social interaction in a group format, and lasted for an optimal amount of time. Therefore, it appears feasible and acceptable to deliver this creative and archeologically informed workshop with few modifications required from its original content, and it could play a role in the context of alternative place-based wellbeing interventions.

While interacting with nature and creativity can enhance wellbeing for various populations (11–14), there is limited research that has examined the intersection of both creative tasks in the context of outdoor natural spaces. Younger generations are leading increasingly urban and technologically-centred lives, and a decline in the level of outdoor experiences and everyday connection with nature has been reported (72). However, existing studies exploring the impact of nature on students have found higher availability and use of outdoor space is associated with improved quality of life and perceived restoration (73, 74). ART and SRT state that natural environments offer a setting where participants are able to restore their directed attention, defined as conscious attention required for cognitive tasks. As this cognitive focus can become fatigued following prolonged mental activity, nature can offer an entirely different setting to gently distract people from the stressors of everyday life (75). Our findings align with previous research, indicating that participants recognized the benefits of outdoor environments. Spending time in natural settings facilitated detachment from the daily attentional demands and stressors. While practical challenges with the outdoor space were reported (e.g., lack of surface space, potential for inclement weather), these challenges did not affect the overall positive perception of the workshop.

In terms of creative activity, participants were offered the opportunity to create a product based on personal preferences and interests, which is crucial for enhanced wellbeing (15). Thematic analysis indicated that participants highly valued the ability to engage in activities they found personally meaningful, with many describing the crafting process as therapeutic and a welcome distraction from daily stress. Evidence supports that a key factor for improved wellbeing is the relevance and meaning incorporated into the crafting process (16, 18, 76). Participants echoed this, noting that the sensory aspects and historical context of the activities added depth and significance to their experience. The entire crafting process, including hands-on engagement and the educational component, was cited as enhancing their sense of satisfaction and enjoyment. This aligns with existing evidence that meaningful crafting activities, encompassing personal relevance and sensory engagement, are imperative for achieving wellbeing (15, 77).

In addition to the creative and environmental aspects of the intervention, participants frequently cited satisfaction with the educational and archaeological content and context of the workshop. Research has indicated that heritage interventions can increase participant knowledge (78, 79), and appreciation of the natural, social, and cultural environment (79). Subsequently, this can facilitate a sense of belonging to the community (78) and an increase in responsible attitudes of care for heritage assets (80). Heritage education develops identity links, whether individually or communally (81), and is linked to various factors including the importance of teachers being innovative and capable to deliver heritage education (79). This aligns with our current findings as skilled facilitation was commonly cited as a key contextual factor of the workshops, whereby the facilitator had extensive archaeological knowledge and expertise. In these senses, this innovative intervention, which combines archaeological-based creative activities with connection to nature, provides valuable insights into new approaches for enhancing capacity and access to alternative place-based wellbeing interventions.

Group-based delivery also appeared to be a key enabler to implementation. This has been shown to be relevant to other creative-based activities that aim to promote wellbeing for a range of populations. For example, previous studies have reported group-based delivery enhanced a sense of belonging (82), improved social relationships (83), social inclusion (84), and the opportunity for learning (85). Moreover, participants reported how shared, yet individual goals, made the workshops socially inclusive. This sense of inclusivity and shared understanding was felt as one of the key contributing factors to wellbeing as it facilitated the establishment of new connections (31). Evidence reports that a crucial component of good mental health is connectedness (86), which is conceptualised as a sense of belonging, perceived sense of support, a sense of perceived closeness to a group, and comfort in discussing problems with others (87). Therefore, providing future workshops in a group format is likely to enhance wellbeing by maximising peer-to-peer support.

Lastly, the majority of participants reported that the length of the workshops was beneficial and offered an ideal balance for educational material at the beginning to provide archaeological context to the crafting, and sufficient time to meaningfully engage with the creative process. Crafting activities are often structured to include an antecedent component prior to the crafting itself, such as art viewing or education (31, 88–90). While this structure has been successfully implemented and positive outcomes are often reported, longer-term heritage crafting activities have the potential to be more impactful, yield sustained mental and cognitive benefits, and offer participants the opportunity to engage in a wider range of crafting activities (89, 91). Therefore, future research should consider moving beyond ‘temporary relief’ provided by a one-off session and incorporate access to comparable workshops over the longer term. Here there are opportunities to exploit growing interest in the role of social prescribing and to evaluate the mental health and wellbeing benefits of outdoor heritage crafting in populations with high vulnerabilities for mental health problems.

### 4.1 Limitations

Generalisability of our findings is limited by several factors. Firstly, our study recruited a convenience sample of student volunteers. While this is a commonly used approach in feasibility studies, students vary as much as the general population both between and within countries, therefore, generalising from students to the general public becomes problematic (92). Secondly, because participants self-selected to join the study, selection bias may influence the findings. For example, it may be possible that only individuals who were interested in craft heritage participated in the study, resulting in the overall positive perception of the workshops. However, there is no ‘one-size fits all’ approach to wellbeing interventions, and personal preferences for activities will inevitably vary depending on personal interests. Lastly, the sample was predominantly comprised of females (68.8%). However, this is a commonly cited finding in research exploring the impact of cultural and crafting activities (76, 93, 94). Despite these limitations, our findings contribute further insight into new approaches that may have the potential to enhance access to alternative wellbeing interventions.

## 5 Conclusion

This study has shown that it is feasible to deliver and evaluate an archaeologically informed outdoor heritage crafting intervention to support wellbeing. The workshops were perceived to be acceptable and highly beneficial among a student volunteer population. Critical to the success of the workshops was the role of a skilled facilitator with an archaeological background, pointing to the relevance of offering participants activities that have heritage value. Furthermore, offering the workshops in an outdoor space with natural features bolstered participants’ positive ratings of the intervention, highlighting the potential to accrue wellbeing benefits from contact with and connection with nature. The added value of undertaking these activities in groups was also key to high levels of engagement, suggesting that the intervention offers opportunities to build social connections and draw on peer-to-peer learning. There is scope to undertake further and definitive evaluation of the mental health and wellbeing benefits of outdoor heritage crafting and respond to policy imperatives to develop preventive approaches to mental health using community and place-based approaches.

## Data Availability

The raw data supporting the conclusions of this article will be made available by the authors, without undue reservation.
