# Supplementary material for: Craftwell: a feasibility and acceptability study of outdoor heritage crafting for wellbeing and mental health
Source: Front Public Health. 2025 Mar 5;13:1556230. doi: 10.3389/fpubh.2025.1556230 (PMC11922075; doi:10.3389/fpubh.2025.1556230)
Supplement: Supplementary file 1 [file Table_1.DOCX]

Supplementary Material

**Supplementary Material 1: Interview topic guide**

**Intro:** Can you tell me a little bit about yourself (i.e., UG/PG, course of study, experience of being a student, any health conditions)

**Part 1: Acceptability, Feasibility and Assessment of Participant Burden**

Research Process

- Can you describe your overall experience of the research process from the initial EOI to participation in the workshop?
  - What did you feel worked well?
  - Were there any difficulties or challenges for you at any stage?

Completion of Pre- and Post-Questionnaires

- Can you describe your experience of completing the questionnaires?
  - What was your experience of Qualtrics compared to the Paper-Based Questionnaires? Any differences/challenges?
  - Were the Questionnaires easy to complete?
  - Were there any questions that were difficult to answer? Why?

Workshop:

- What workshop did you take part in?
  - Did you experience any difficulties with locating the workshop?
- How would you describe your experience of the workshop?
- If there was anything you would change about the workshop, what would it be? More/less? Longer/shorter?

**Part 2: Participants Experiences**

Motivation:

- Why did you sign up for this workshop?
- What was it about this activity that interested you?
- What did you like about this activity?
- Was there anything about this activity you did not like/found challenging?
- How does this compare to other activities you have taken part in at the university?
  - Have you participated in any other activities at the University to support your health and wellbeing?

Social dynamics:

- Did you attend on your own or did you come with friends?
- Did this influence your decision/your experience of participating in the workshop?

Crafting experience:

- Do you participate in any crafting activities already? If Yes, what type of activities?
- How did engaging in this workshop activity make you feel?

Heritage:

- How important to you were the heritage/archaeological aspects of this workshop? Why?
  - Did you have an interest in history/prehistory already?
  - Can you describe the significance for you of the archaeological/historical aspect of the workshops that appealed/was of importance to you?
  - Is there anything about connecting with the past that is important for you? Why?
- Would the activity be of interest to you without the ‘archaeology bit’? Why/Why not?

Environment:

- How important for you was the outdoor space for this activity? Why/Why was it not important?
- Was there anything about the outdoor space that you specifically liked/enjoyed?
- Was there anything about the outdoor space that you did not like/found challenging?
  - Safety, comfort, temperature?

Mental health / wellbeing:

- How would you describe your overall experience of taking part in the activity?
  - Was this a positive or negative experience overall?
- Were there any benefits to you in participating in this activity?
  - Mental health and wellbeing?
  - Social?
  - Other?

Engagement:

- Did you discover/learn anything new from participating in the workshop?
  - Did you discover anything about yourself/outdoors/heritage/archaeology?
- Has anything changed for you as a result of taking part in the workshop?
  - Has it encouraged you to find out more about the outdoors/heritage/archaeology/crafting/become more curious?

Follow up engagement for participants in workshop:

- Since you have participated in the workshop, have you used the pot/beads you made in any way?
- If so, in what way?
- If not, why?
- If participants did not collect pots, was there any specific reason(s) you did not collect your pot?

Final Question:

- Is there anything else you would like to add which you think is important, that I have not already asked?
